# HYAL3 as a potential novel marker of BLCA patient prognosis

**DOI:** 10.1186/s12863-022-01070-w

**Published:** 2022-08-09

**Authors:** Jun-peng Liu, Yu-tong Fang, Yi-fan Jiang, Hao Lin

**Affiliations:** 1 Department of Urology, The Second Affiliated Hospital of Shantou University Medical College, Shantou, 515041 Guangdong Province China; 2 The Breast Center, Cancer Hospital of Shantou University Medical College, Shantou, 515041 Guangdong Province China

**Keywords:** Bladder cancer, HYAL3, Immune cells, TCGA, Bioinformatic analysis

## Abstract

**Background:**

It has been previously demonstrated that hyaluronan (HA) potentially regulates the initiation and propagation of bladder cancer (BLCA). *HYAL3* encodes hyaluronidase and is a potential therapeutic target for BLCA. We aimed to explore the role that HYAL3 plays in BLCA pathogenesis.

**Methods:**

*HYAL3* expression in BLCA specimens was analyzed using The Cancer Genome Atlas (TCGA) database and the Gene Expression Omnibus (GEO) cohort as well as confirmed in cell lines and The Human Protein Atlas. Then, associations between *HYAL3* expression and clinicopathological data were analyzed using survival curves and receiver-operating characteristic (ROC) curves. The functions of HYAL3 were further dissected using Kyoto Encyclopedia of Genes and Genomes (KEGG) pathway analysis and the protein–protein interaction network. Finally, we harnessed the Tumor IMmune Estimation Resource and Gene Expression Profiling Interactive Analysis to obtain correlations between *HYAL3* expression, infiltrating immunocytes, and the corresponding immune marker sets.

**Results:**

*HYAL3* expression varied greatly between many types of cancers. In addition, a higher *HYAL3* expression level predicted a poor overall survival (OS) in both TCGA-BLCA and GEO gene chips (*P* < 0.05). HYAL3 also exhibited an acceptable diagnostic ability for the pathological stage of BLCA (area under the receiver-operating characteristic curve = 0.769). Furthermore, *HYAL3* acted as an independent prognostic factor in BLCA patients and correlated with the infiltration of various types of immunocytes, including B cells, CD8^+^ T cells, cytotoxic cells, T follicular helper cells, and T helper (Th) 2 cells.

**Conclusion:**

*HYAL3* might serve as a potential biomarker for predicting poor OS in BLCA patients and correlated with immunocyte infiltration in BLCA.

**Supplementary Information:**

The online version contains supplementary material available at 10.1186/s12863-022-01070-w.

## Background

Bladder cancer (BLCA) ranks among the 10 most common malignancies, accounting for nearly 54,900 incident cases and 200,000 deaths in 2018 [1]. Nonmuscle invasive bladder cancer (NMIBC) is the most common subtype, comprising about 75% of all cases. With the characteristics of a high replication rate and a high risk of progression to invasive BLCA, patients with an NMIBC of ≥3 cm or grade G3 on pathology have an unfavorable prognosis [2]. The pathogenesis of BLCA is complex, and the mechanism responsible for its development remains unclear. Prior studies have suggested that the pathological stage, the neutrophil-to-lymphocyte ratio, the microRNA (miRNA) let-7 g [3], the lymphocyte-to-monocyte ratio, the platelet-to-lymphocyte ratio [4], the long noncoding RNA LINC00641 [1], the protein Ki-67 [5], and the serum cholinesterase level can be used to predict the prognosis of BLCA patients.

Extracellular matrix (ECM) alteration is closely associated with tumor invasion and progression [6]. In addition, dysregulated ECM is associated with the epithelial-to-mesenchymal transition involving stem cells in cancer. The ECM also regulates tissue metabolism and facilitates the development and progression of different cancers, including BLCA [7]. Hyaluronic acid (HA) is a glycosaminoglycan mainly associated with the ECM. While HA does not induce cellular transformation, it supports other important tumor phenotypes, such as proliferation, migration, resistance to proapoptotic stimuli, and epithelial-to-mesenchymal transition [8].

In previous reports, HA was found to be a reliable tumor marker in patients with urothelial carcinoma [9]. Hyaluronidase (HYAL) is an endogenous glycosidase that degrades HA through the restrictive digestion site at the β-1,4-glycosidic bond between D-glucuronic acid and N-acetylglucosamine. This is achieved by breaking the β-1,4-glycosidic bond between 2-acetyl-D-deoxygenation-D-glucose and D-glucuronic acid [10]. HYAL was first discovered by Duran-Reynals [11]. Through genomic sequencing analyses, researchers have identified multiple HYAL family members, including HYAL1, HYAL2, HYAL3, HYAL4, PH20, and HYALP1. *HYAL3* is located on human chromosome 3p21.3 [12, 13]. The regulated turnover of HA plays a critical role in many biological processes, including cellular proliferation, migration, and differentiation. Although an association between HYAL3 and tumor development has been reported [14], the mechanism through which HYAL3 regulates tumor phenotypes remains unknown.

According to previous studies, tumor-infiltrating immunocytes, including B, T, CD8^+^ T cells, and others, play an important role in regulating the balance between antitumor immunity and immune escape in BLCA [15–17]. To date, none of the known biomarkers can accurately predict the therapeutic response to immune checkpoint inhibitors in patients with BLCA. However, several reports have shown that cisplatin-based combination chemotherapy might increase CD8^+^ T cell infiltration and programmed death ligand 1 expression while decreasing the number of immune-suppressing cells [18]. Therefore, it is important to explore the potential mechanisms through which tumor-infiltrating immunocytes regulate the therapeutic responses to chemotherapy and immune checkpoint inhibitors in BLCA.

In the current study, we analyzed the relationships between *HYAL3* expression levels, clinical features, and overall survival (OS) in patients with BLCA, utilizing The Cancer Genome Atlas (TCGA), Gene Expression Omnibus (GEO), and the Human Protein Atlas databases. This approach was followed by using the Metascape website to enable Kyoto Encyclopedia of Genes and Genomes (KEGG) and Gene Ontology (GO) enrichment analyses and the Search Tool for the Retrieval of Interacting Genes/Proteins (STRING)-enabled analysis of the HYAL3-associated protein–protein interaction network. We further used the Tumor IMmune Estimation Resource (TIMER) and the Gene Expression Profiling Interactive Analysis (GEPIA) databases to analyze the associations between the *HYAL3* expression level, the type of infiltrating immunocytes, and their corresponding gene marker sets.

## Methods

### Data source

All TCGA expression datasets from RNA-seq were downloaded from the TCGA website (https://portal.gdc.cancer.gov/). Only cancer datasets including paired samples for an accurate identification of differentially expressed genes were incorporated into our study. No blood samples were included into the analysis as normal samples. A total of 18 cancer datasets matched these criteria. We employed the “RUVg” function in the package RUVSeq (v3.8) to correct the batch effect in the RNA-seq datasets [19]. The identification of differentially expressed genes was performed by the “glmTreat” function in the package edgeR (v3.24.0) [20]. Genes with a count-per-million ≥0.1 in the normal samples were defined as expressed genes. The normalized expression value of trimmed mean of M-values was also generated by the “edgeR” package. We used the above package in R version 3.6.3.

### GEO database and the human protein atlas

BLCA-related profiles were obtained from the GEO database (http://www.ncbi.nlm.nih.gov/geo/). Data that met the following criteria were selected: (I) studies including at least 20 samples and (II) examination of mRNA expression in both cancer tissue and adjacent normal tissue from BLCA patients. Finally, GSE31684 was selected as our validation cohort. Studies without useful data for analysis were excluded. Differentially expressed genes between BLCA and normal tissue samples were ranked by the Robust Multi-Array Average and Linear Models and annotated by converting the different probe IDs to gene IDs [21].

### TIMER and GEPIA databases

TIMER (Version 2.0) was established to explore the abundance of immunocyte infiltration in different tumors. We used TIMER to assess the relationship between *HYAL3* and six types of infiltrating immunocytes (CD8^+^ T cells, CD4^+^ T cells, B cells, dendritic cells (DCs), macrophages, and neutrophils) in BLCA via gene modules. Expression dispersion maps were created between a pair of custom genes for gastric cancer, and the statistical significance of the correlation and estimation by Spearman analysis were determined by the correlation module. The level of gene expression was shown as log2 RSEM (RNA-Seq by Expectation-Maximization).

GEPIA (http://gepia.cancer-pku.cn/index.html) is an online database consisting of more than 8000 types of tumors and normal tissues from the TCGA and the Genotype-Tissue Expression (GTEx) databases. We used these data to explore the association between *HYAL3* expression and multiple immunologic marker datasets. The Spearman method was used to determine the correlation coefficient, and the median value of the *HYAL3* expression was used as a cutoff to distinguish high expression from low expression.

### Cell lines

The human BLCA cell lines T24 and 5637 as well as the noncancerous urothelial cell line SV-HUC were purchased from the Shanghai Institute of Cell Biology, Chinese Academy of Sciences. These cells were cultured in RPMI 1640 (Procell Life Science & Technology, China) and Dulbecco’s modified Eagle’s medium (Procell Life Science & Technology, China) supplemented with 10% fetal bovine serum (Ausbian Corporation, Australia) and 1% penicillin/streptomycin (Beyotime, Shanghai Biyuntian Biology Technology, China). The cells were maintained at 37 °C in a CO_2_ incubator. When the cells reached 80% confluence, the cells were trypsinized and passaged at a 1:3 ratio.

### qRT-PCR

Total cellular RNAs were extracted using TRIzol reagent (Invitrogen, USA), according to the manufacturer’s instruction. The quantity of RNA was calculated based on the absorbance at 260 nm detected by a NanoDrop 2000 spectrophotometer. An absorbance ratio (260 nm/280 nm) between 1.8 and 2.0 was considered as good purity RNA and used for further experiments. Samples of RNA (2 μg) were transcribed into cDNAs with the PrimeScript RT reagent Kit with gDNA Eraser (TAKARA Corporation) in a final volume of 50 μL. Specific cDNAs were amplified with SYBR® Green Master Mix (TAKARA Corporation) utilizing an ABI 7500 Real-Time PCR System (Applied Biosystems, Foster City, CA, USA). The reaction conditions were as follows: 95 °C for 2 min; followed by 95 °C for 30 s, and 40 cycles of 95 °C for 5 s and 60 °C for 34 s. The results were analyzed by using the 2 − ΔΔCT relative quantitative method, with GAPDH as an internal control.

Gene-specific primers for *HYAL3* and the reference *GAPDH* were designed using the National Center for Biotechnology Information Primer-Blast Tool (https://www.ncbi.nlm.nih.gov/tools/primer-blast/). All reactions were performed in triplicate, and their melting curves were analyzed to confirm their specificity and accuracy. The expression levels of *HYAL3* were normalized to the levels of *GAPDH*. The sense and antisense primer sequences for *HYAL3* and *GAPDH* were as follows: *HYAL3*, forward 5′-GGCCAACGTTGTCGGACCGAT-3′, reverse 5′-CAGCATGGCAGCGGCCGGTATAG-3′; and *GAPDH*, forward 5′-CAGGAGGCATTGCTGATGAT3′, reverse 5′-GAAGGCTGGGGCTCATTT-3′.

### Univariate and multivariate cox regression analyses

To determine the influence of HYAL3 on the outcome of patients with BLCA, we used univariate Cox regression analysis to examine the association between *HYAL3* and OS in the TCGA-BLCA cohort. Then, we further used multivariate Cox regression analysis to determine whether *HYAL3* could independently predict the prognosis of patients with BLCA. The confidence interval (CI) was set at 95%, and a *P*-value < 0.05 was considered to indicate a significant difference in the statistical analyses.

### Survival curves and receiver-operating characteristic (ROC) curves

We constructed Kaplan–Meier survival curves according to the expression levels of *HYAL3* to investigate whether *HYAL3* expression affected the outcomes of patients with BLCA. Meanwhile, to evaluate the predictability of *HYAL3* expression for BLCA prognosis, we incorporated the clinical and pathological data from TCGA-BLCA and *HYAL3* expression to generate ROC curves.

### Linkedomics database

The Linkedomics Database (http://www.linkedomics.org) includes different sequencing data from 32 cancers in the TCGA and online clinical databases. The *HYAL3*-related genes were analyzed statistically using Pearson’s correlation coefficient, and the data were presented in volcano plots, heat maps, or scatter plots. The results showed the genes exhibiting the closest association with *HYAL3* in TCGA-BLCA.

### Protein–protein interaction network

We also used the STRING website (https://string-db.org/) to predict the proteins that interacted with *HYAL3*. We inputted *HYAL3* and then set the confidence score at > 0.4 for significance.

### GO and KEGG enrichment analyses

We used the Metascape website (https://metascape.org/gp/index.html) to conduct GO and KEGG enrichment analyses based on the top 200 genes related to *HYAL3* in BLCA. GO consists of three domains: molecular function (MF), cellular component (CC), and biological process (BP). Terms with a *P*-value< 0.01, a minimum count of 3, and an enrichment factor > 1.5 were collected and grouped into clusters based on their membership similarities.

### Statistical analysis

Each experiment was repeated at least three times. Data are expressed as the mean ± standard deviation. Differences between and among groups were compared using the independent-samples T test for qualitative variables. All statistical tests were two-sided, and statistical significance was defined as *P* < 0.05.

## Results

### Patient characteristics

**Fig. 1:**
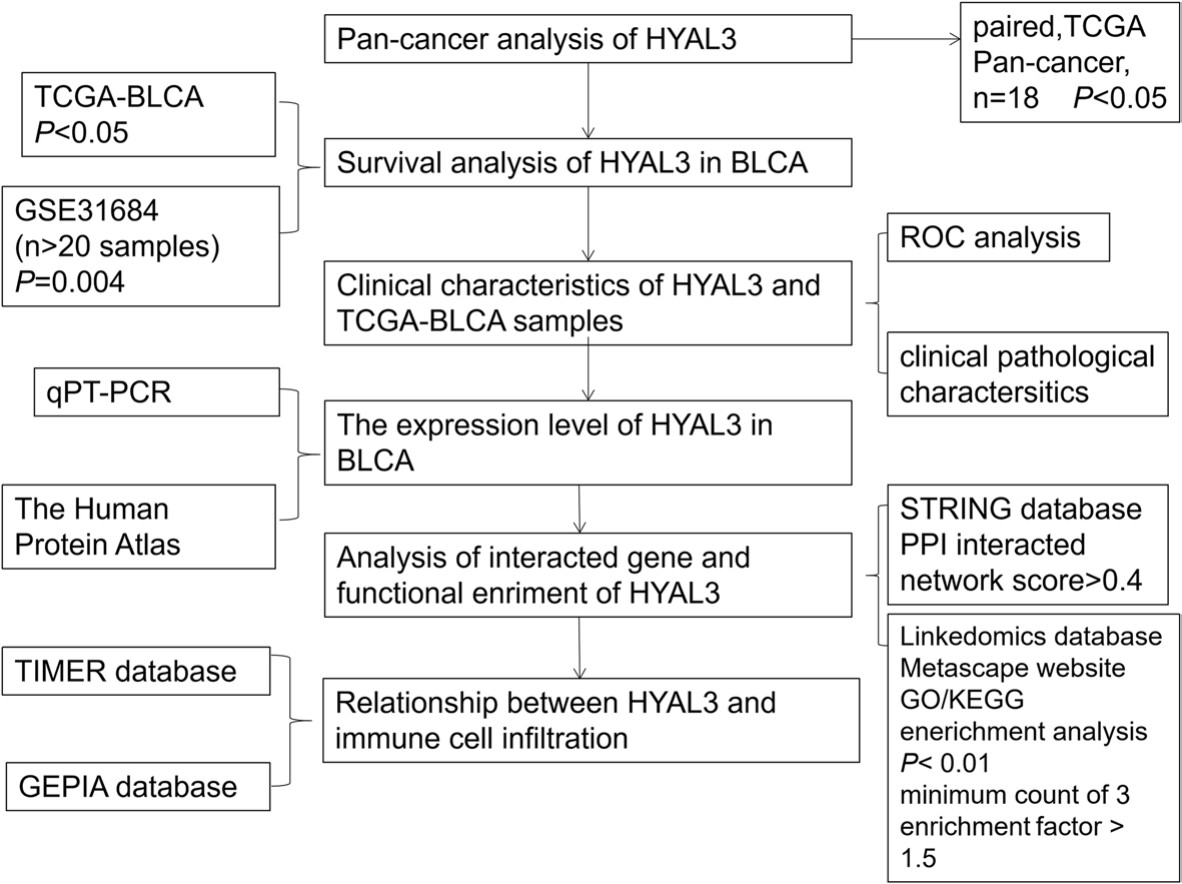
The flow chart of the methodologies used in this study

The flow chart of the methodologies used in this study is presented in Fig. 1. Our study included data from RNA sequencing and information related to the patient prognosis from 414 BLCA samples and 40 normal tissues in the TCGA and the GTEx databases. All patients were divided into two groups according to the *HYAL3* expression levels. Table 1 summarizes all of the clinical and pathological data of the BLCA patients, including the tumor–lymph node–metastasis (TNM) stage, pathological stage, sex, histological stage, subtype, smoking status, OS, lymphovascular invasion status, and age.

**Table 1:** Clinical and pathological data of patients included in TCGA-BLCA

Characteristic	Low expression of *HYAL3*	High expression of *HYAL3*	*P*
n	207	207	
T stage, *n* (%)			0.504
T1	4 (1.1%)	1 (0.3%)	
T2	56 (14.7%)	63 (16.6%)	
T3	102 (26.8%)	94 (24.7%)	
T4	31 (8.2%)	29 (7.6%)	
N stage, *n* (%)			0.432
N0	128 (34.6%)	111 (30%)	
N1	23 (6.2%)	23 (6.2%)	
N2	36 (9.7%)	41 (11.1%)	
N3	6 (1.6%)	2 (0.5%)	
M stage, *n* (%)			0.962
M0	103 (48.4%)	99 (46.5%)	
M1	5 (2.3%)	6 (2.8%)	
Pathologic stage, *n* (%)			0.143
Stage I	4 (1%)	0 (0%)	
Stage II	59 (14.3%)	71 (17.2%)	
Stage III	75 (18.2%)	67 (16.3%)	
Stage IV	67 (16.3%)	69 (16.7%)	
Sex, *n* (%)			0.264
Female	60 (14.5%)	49 (11.8%)	
Male	147 (35.5%)	158 (38.2%)	
Age, *n* (%)			0.275
≤ 70	123 (29.7%)	111 (26.8%)	
> 70	84 (20.3%)	96 (23.2%)	
Histologic grade, *n* (%)			0.662
High Grade	196 (47.7%)	194 (47.2%)	
Low Grade	9 (2.2%)	12 (2.9%)	
Subtype, *n* (%)			0.623
Non-Papillary	140 (34.2%)	135 (33%)	
Papillary	64 (15.6%)	70 (17.1%)	
Smoker, *n* (%)			0.894
No	56 (14%)	53 (13.2%)	
Yes	146 (36.4%)	146 (36.4%)	
OS event, *n* (%)			0.003
Alive	131 (31.6%)	100 (24.2%)	
Dead	76 (18.4%)	107 (25.8%)	
Lymphovascular invasion, *n* (%)			1.000
No	67 (23.7%)	63 (22.3%)	
Yes	78 (27.6%)	75 (26.5%)	
Age, median (IQR)	68 (59.5, 75)	69 (61.5, 76.5)	0.112

### Higher HYAL3 expression levels in tumor samples than in normal tissues

**Fig. 2:**
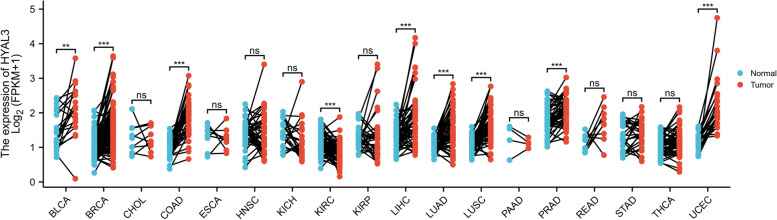
The mRNA-sequencing expression levels of *HYAL3* in the pan-cancer panel

**Fig. 3:**
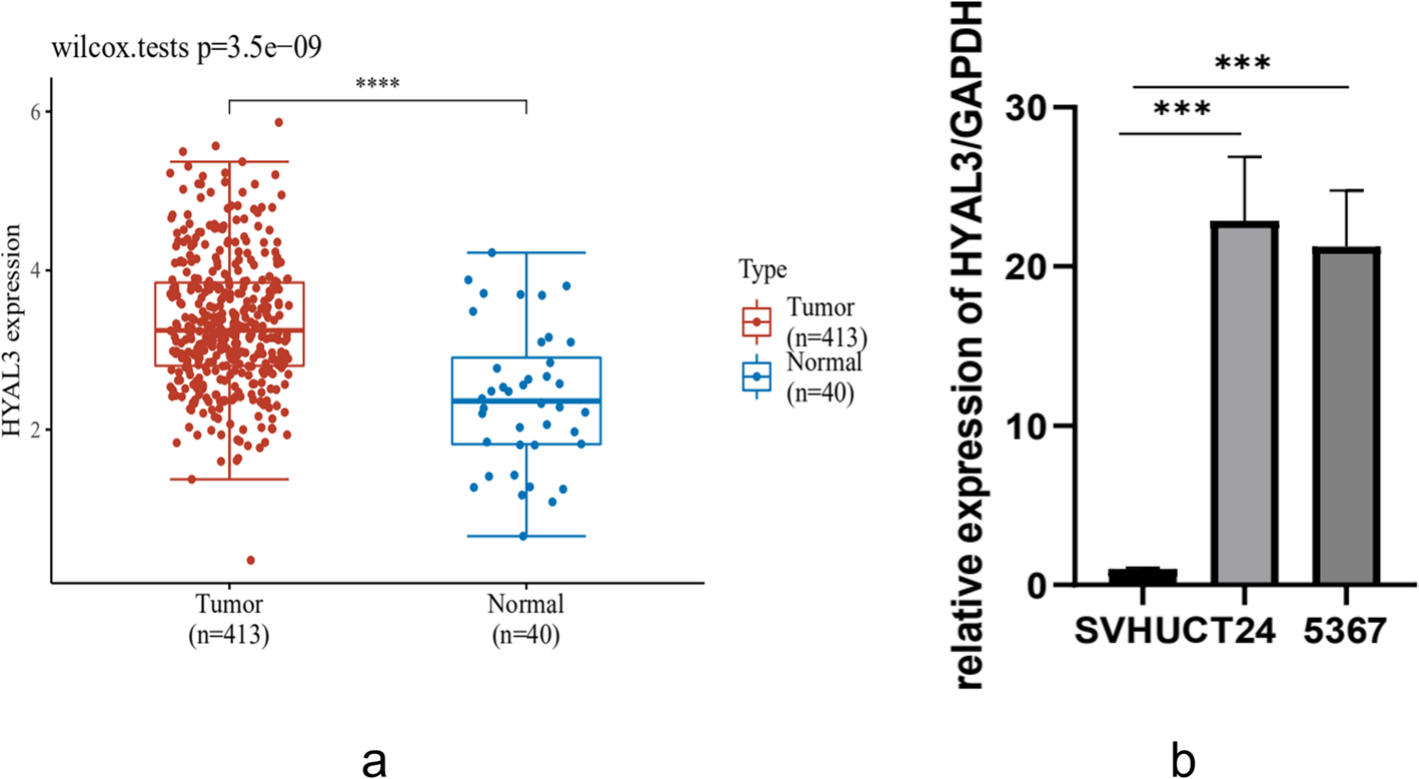
Expression levels of *HYAL3* between bladder cancer (BLCA) and normal tissues. **a** Expression levels of the *HYAL3* in TCGA-BLCA and normal tissues in the TCGA and the GTEx databases. **b** The mRNA expression levels of *HYAL3* in the 5637, T24, and SVHUC cell lines

**Fig. 4:**
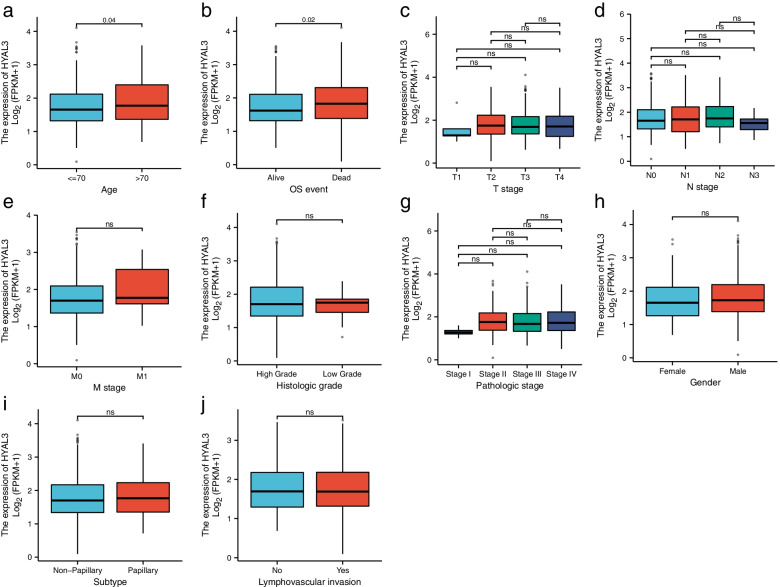
The correlation between *HYAL3* expression and clinical and pathological parameters in bladder cancer (BLCA) patients. The correlation between *HYAL3* expression and age (**a**), OS event (**b**), TNM stage (**c**–**e**), histological grade (**f**), pathological grade (**g**), sex (**h**), subtype (**i**), or lymphovascular invasion (**j**)

To explore the role of HYAL3 in the development of BLCA, we analyzed the mRNA-sequencing expression levels of *HYAL3* in various types of cancer (Fig. 2). The results indicated that *HYAL3* might serve as an oncogene in the development of cancers including BLCA. We noted that the expression levels of *HYAL3* were significantly higher in BLCA than in normal tissues in the TCGA and the GTEx databases (*P* = 3.5 e− 09) (Fig. 3a). These findings were also validated by the qRT-PCR assays using the BLCA cell lines (Fig. 3b). Furthermore, we validated the expression levels of HYAL3 in BLCA using the Human Protein Atlas. We found that the expression levels of HYAL3 in BLCA tissues were upregulated compared with those in normal tissues (Table 2). However, when the associations between *HYAL3* expression and clinicopathological parameters in BLCA patients were compared, we found that the *HYAL3* mRNA expression levels were higher in those aged > 70 years old and in those who died compared with the other patients (*P* = 0.02 and 0.04, respectively; Fig. 4a–b). In contrast, the *HYAL3* mRNA levels did not differ according to the TNM stage, histological grade, pathological grade, sex, subtype, or lymphovascular invasion (*P* > 0.05) (Fig. 4c–j).

**Table 2:** Clinical data and relative scores of immunohistochemistry results from the Human Protein Atlas database

Protein	Tissue	Histological type	Age	Sex	Location	Quantity	Intensity	Relative IHC score
HYAL3	Urothelial carcinoma	Urinary bladder	89	Male	membranous nuclear	> 75%	Moderate	8
HYAL3	Urothelial carcinoma	Urinary bladder	89	Male	Cytoplasmic/membranous nuclear	> 75%	Moderate	7
HYAL3	Urothelial carcinoma	Urinary bladder	63	Female	Cytoplasmic/membranous	75–25%	Moderate	6
HYAL3	Urothelial carcinoma	Urinary bladder	63	Female	Cytoplasmic/membranous	75–25%	Moderate	6
HYAL3	Normal tissue	Urinary bladder	55	Male	Nuclear	75–25%	Weak	2
HYAL3	Normal tissue	Urinary bladder	51	Male	Nuclear	75–25%	Weak	0

The above results were cited from The Human Atlas (https://www.proteinatlas.org/)

*IHC* Immunohistochemistry

### Higher HYAL3 mRNA expression correlates with a shorter OS in BLCA patients

**Fig. 5:**
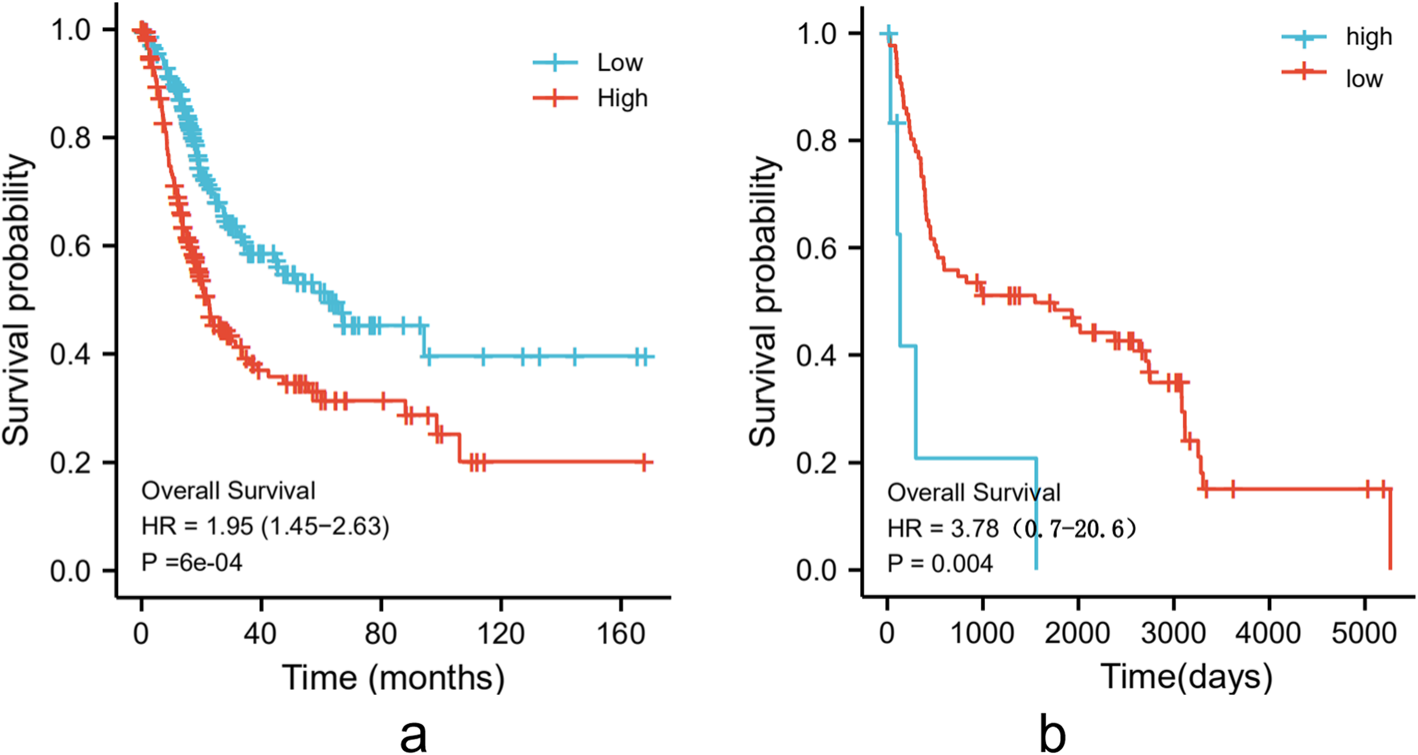
The Kaplan–Meier survival curves according to high or low *HYAL3* expression in TCGA-BLCA (**a**) and GSE31684 (**b**)

**Fig. 6:**
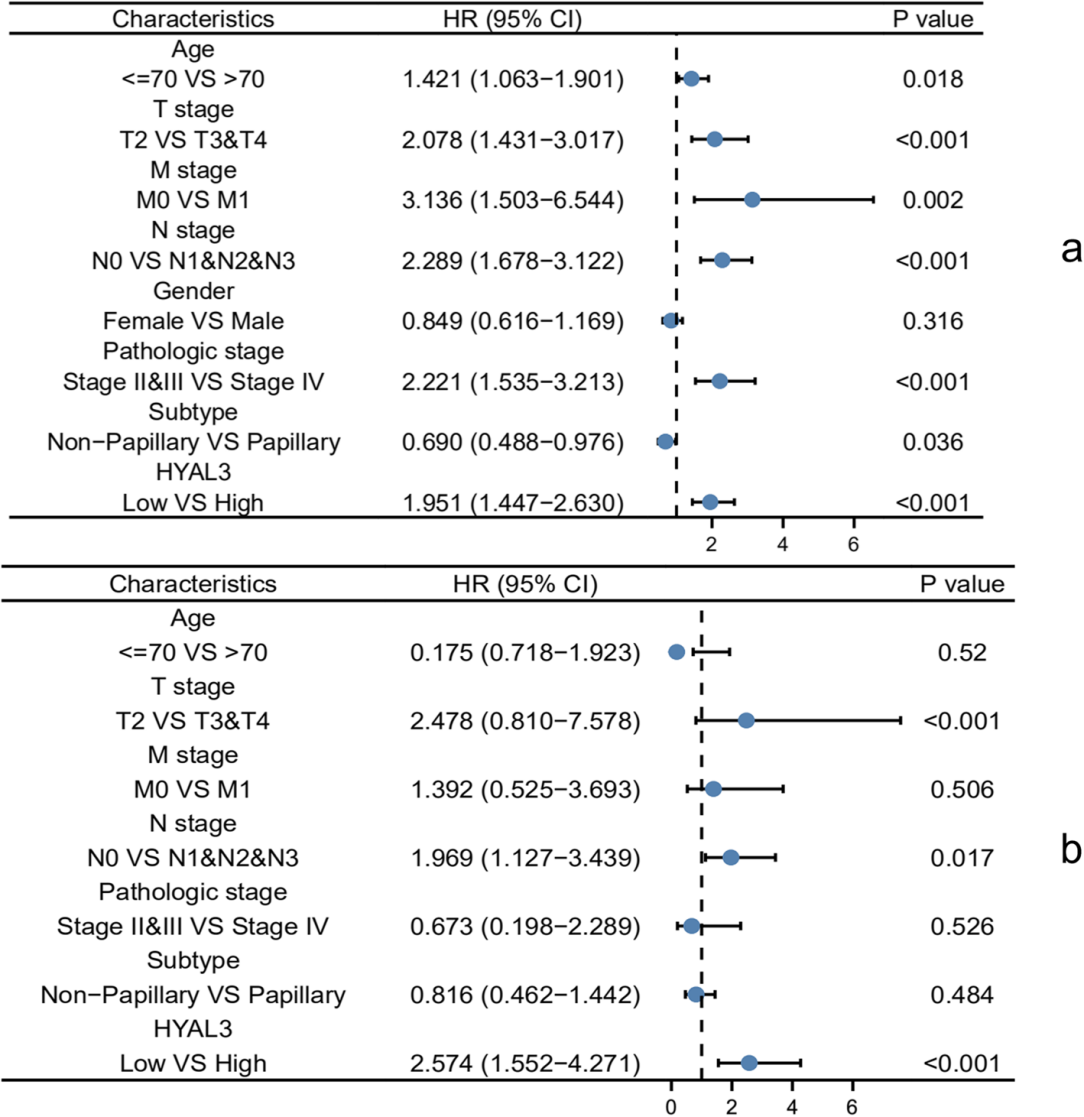
The univariate (**a**) and multivariate (**b**) Cox regression analyses of *HYAL3* expression and clinical data

To explore the influences of HYAL3 on the OS of BLCA patients, we constructed Kaplan–Meier survival curves according to the expression levels of *HYAL3* to investigate whether *HYAL3* expression affected the outcomes of patients with BLCA. The results showed that BLCA cases with lower *HYAL3* mRNA expression levels had a significantly longer OS in the TCGA-BLCA cohort (*P* < 0.001; Fig. 5a), and similar findings were also observed in the GSE31684 cohort (*P* = 0.004; Fig. 5b). According to univariate Cox regression analysis, we found that the TNM stage, age, subtype, pathological stage, and *HYAL3* expression level were associated with the OS of the BLCA patients. Multivariate Cox regression analyses indicated that the expression level of *HYAL3* could be an independent prognostic factor for BLCA patients (Fig. 6).

### HYAL3 as a potential biomarker for predicting the pathological stage of BLCA

**Fig. 7:**
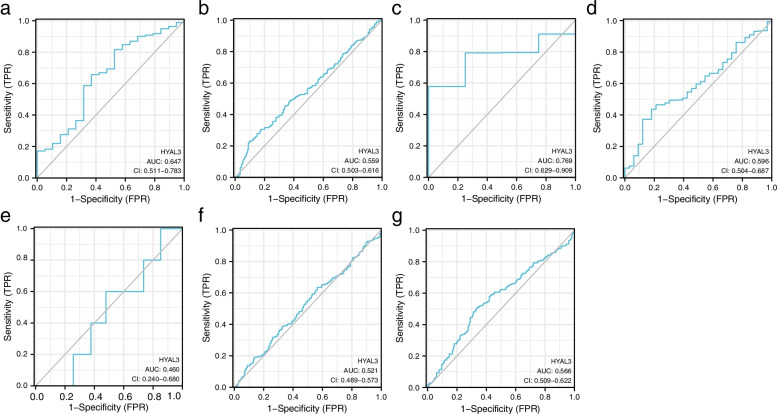
ROC curves of *HYAL3* expression and different clinical data of bladder cancer (BLCA) patients. ROC curves indicating the correlation of *HYAL3* expression with status events (**a**), age (**b**), pathological stage (**c**), T stage (**d**), M stage (**e**), N stage (**f**), and OS (**g**)

We constructed Kaplan–Meier survival curves according to the expression levels of *HYAL3* and used ROC curves for quantifying predictive efficacy (Fig. 7a–g). When the *HYAL3* expression was used to predict BLCA presence/absence, the AUC was 0.647 (95% CI: 0.511–0.783). If the cutoff value was set at 1.238, the sensitivity and specificity were 81.6 and 71.4%, respectively (Fig. 7a). When the *HYAL3* expression was used to predict the patient’s age, the AUC was 0.559 (95% CI: 0.511–0.783). If the cutoff value was set at 2.525, the sensitivity and specificity were 22.8 and 90.6%, respectively (Fig. 7b). When the *HYAL3* expression was used to predict the pathological stage, the AUC was 0.769 (95% CI: 0.629–0.909). If the cutoff value was set at 1.599, the sensitivity and specificity were 57.8 and 99.5%, respectively (Fig. 7c). When the *HYAL3* expression was used to predict the T stage, the AUC was 0.596 (95% CI: 0.504–0.587). If the cutoff value was set at 1.57, the sensitivity and specificity were 43.6 and 81.8%, respectively (Fig. 7d). When the *HYAL3* expression was used to predict the M stage, the AUC was 0.460 (95% CI: 0.240–0.680). If the cutoff value was set at 1.221, the sensitivity and specificity were 19.8 and 99.5%, respectively (Fig. 7e). When the *HYAL3* expression was used to predict the N stage, the AUC was 0.521 (95% CI: 0.469–0.573). If the cutoff value was set at 1.336, the sensitivity and specificity were 63.5 and 43.5%, respectively (Fig. 7f). When the HYAL3 expression was used to predict the OS, the AUC was 0.566 (95% CI: 0.509–0.622). If the cutoff value was set at 1.826, the sensitivity and specificity were 50.8 and 66.2%, respectively (Fig. 7g). The above results suggest that HYAL3 might be a potential biomarker for predicting the pathological stage of BLCA.

### HYAL3 as a tumorigenesis facilitator through interacting with proteins related to immune regulation

**Fig. 8:**
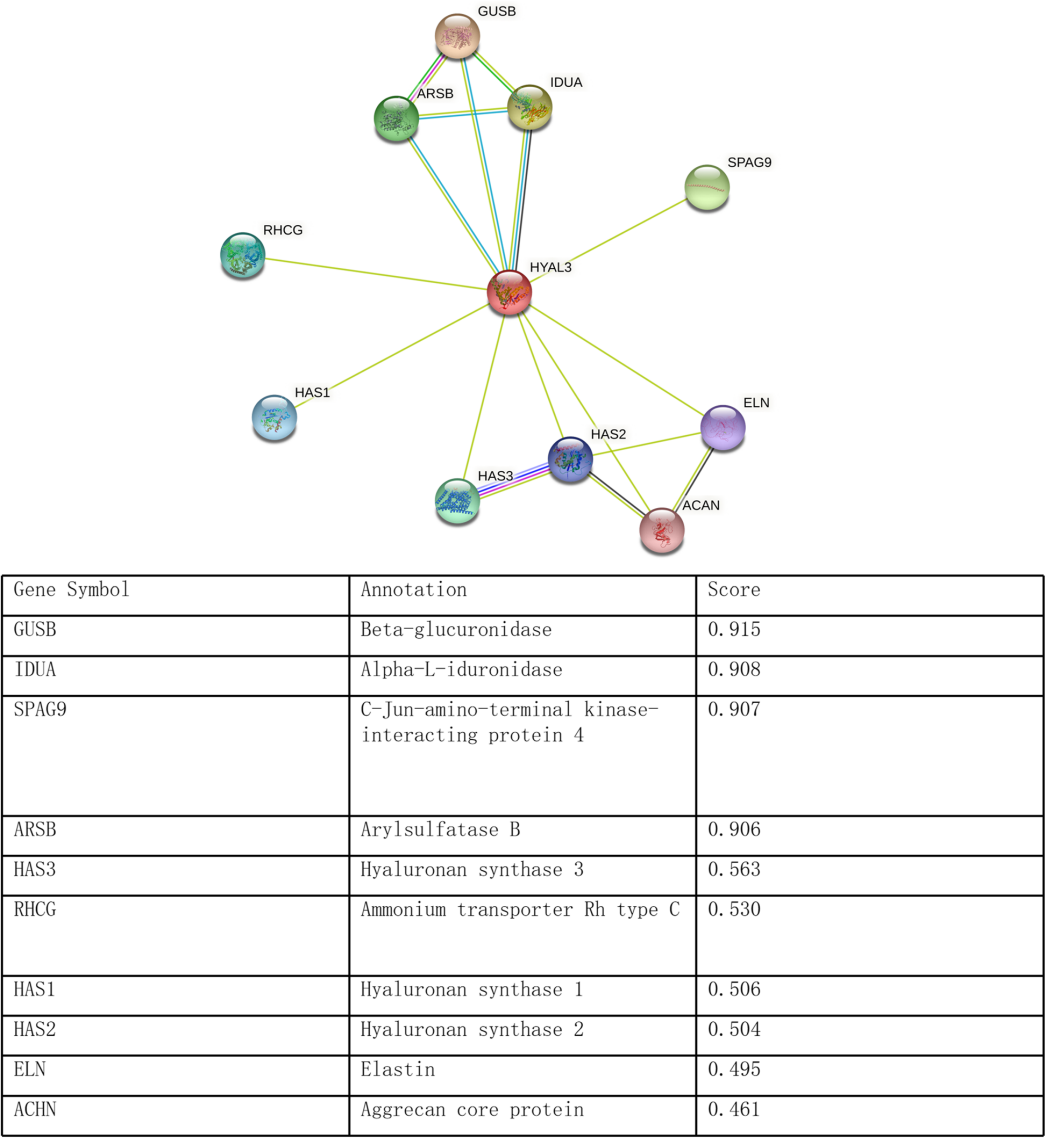
The protein–protein interaction network of HYAL3

To further realize the functions of HYAL3, we used the STRING website (https://string-db.org/) to predict the proteins that interacted with HYAL3. The top 10 interacting proteins and their gene names, annotations, and scores are listed in Fig. 8, including GUSB, RHCG, ARSB, SPAG9, IDUA, ELN, HAS2, HAS1, ACHN, and HAS3. Figure 8 presents the interactions between HYAL3 and 10 target proteins with the highest prediction score. SPAG, IDUA, and ELN play vital roles in the regulation of the immune response. These findings indicate that HYAL3 might promote tumorigenesis by regulating the immune system.

### HYAL3 potentially regulates tumor differentiation and growth

**Fig. 9:**
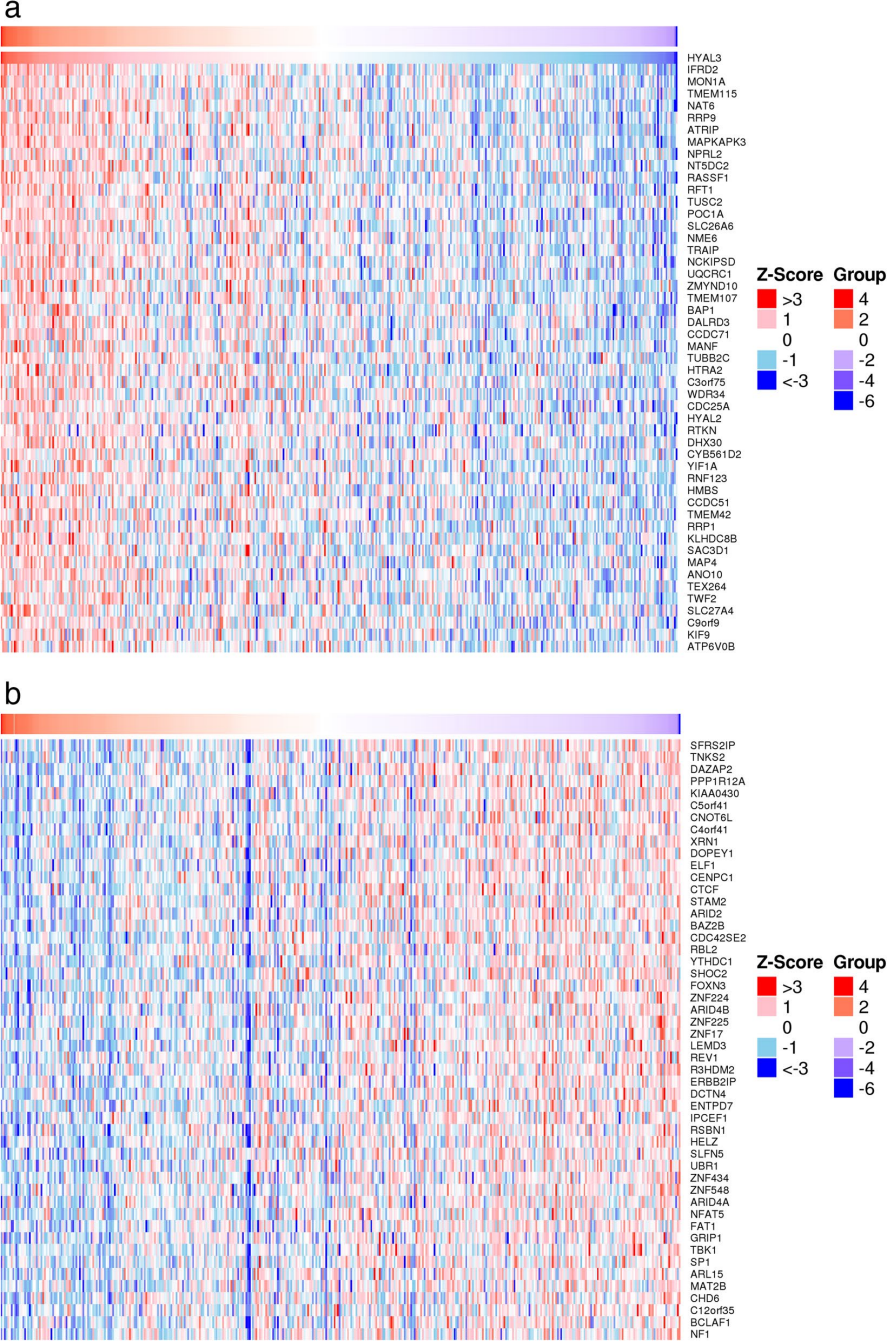
Heat maps of genes negatively (**a**) or positively (**b**) associated with HYAL3 expression

**Fig. 10:**
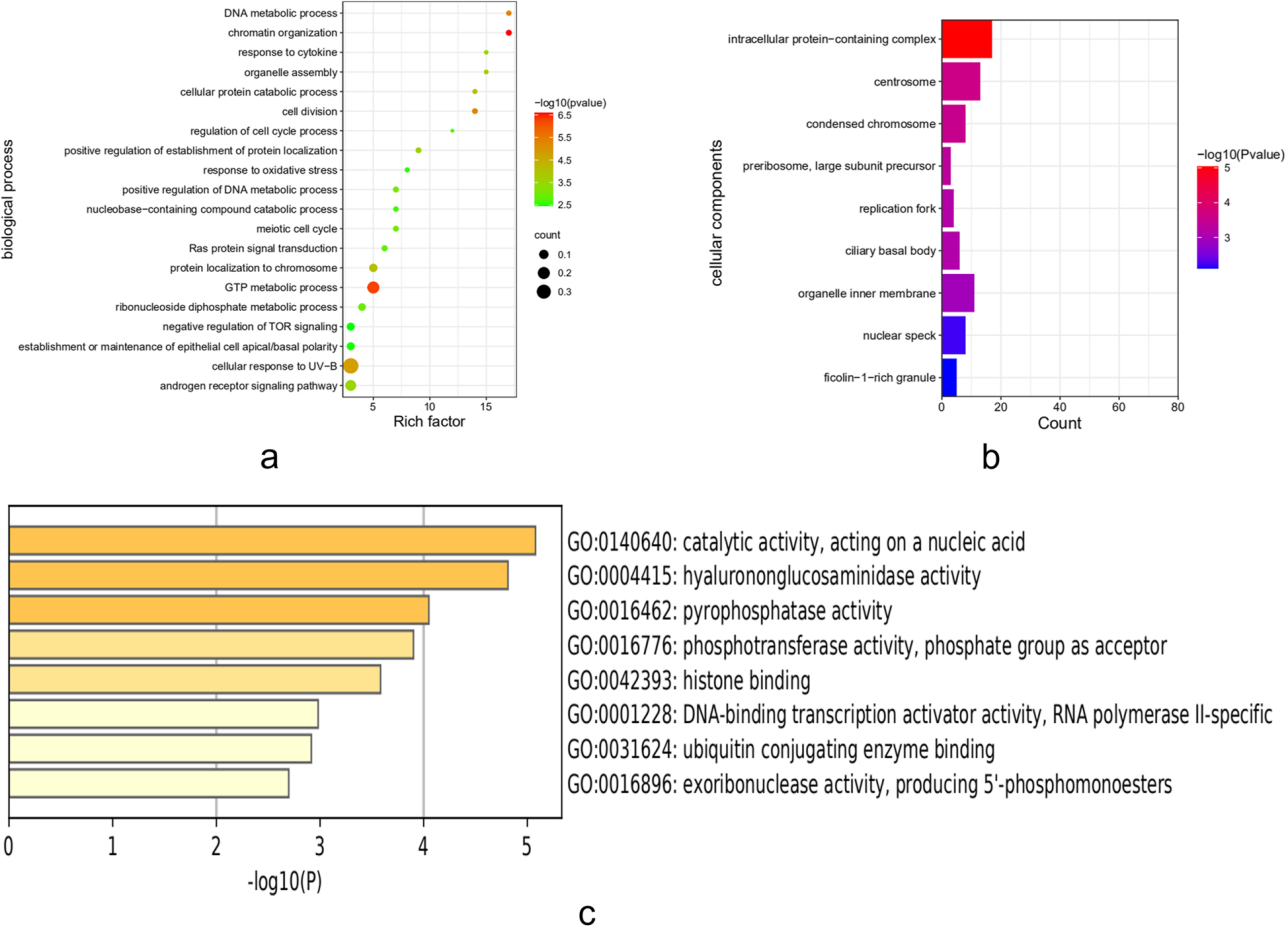
GO functional enrichment analysis of *HYAL3*. **a** The results of enrichment in GO biological processes. **b** The results of enrichment in GO cellular components. **c** The results of enrichment in GO molecular functions

To explore the downstream target of HYAL3, the Linkedomics Database (http://www.linkedomics.org) was used to predict the targets of HYAL3. The HYAL3-related genes were analyzed statistically using Pearson’s correlation coefficient and presented in heat maps. The results showed 200 genes exhibiting the closest association with HYAL3 in TCGA-BLCA (Supplementary Table 1). Heat maps of genes negatively (Fig. 9a) or positively (Fig. 9b) associated with HYAL3 expression are shown in Fig. 10.

**Fig. 11:**
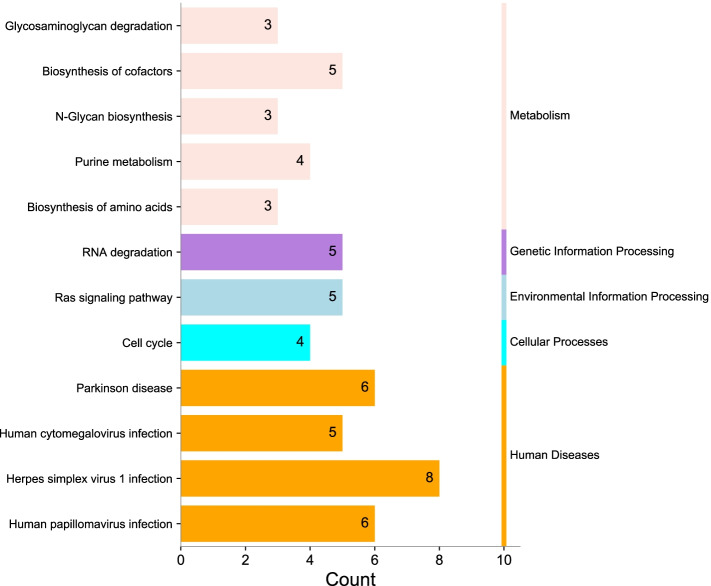
KEGG pathway enrichment analysis of *HYAL3*. **a** The results of KEGG pathway enrichment analysis are divided into five parts. **b** The results of KEGG pathway enrichment analysis

We used the Metascape website to conduct GO and KEGG enrichment analyses based on the top 200 genes related to HYAL3 in BLCA. The GO enrichment analysis results are listed in Fig. 10. The enriched biological processes mainly involved in tumor growth included chromatin organization, GTP and DNA metabolic processes, cell division, cellular response to UV-B, cellular protein catabolic process, positive regulation of the DNA metabolic process, ribonucleoside diphosphate metabolic process, regulation of the cell cycle process, response to oxidative stress, and the establishment or maintenance of epithelial cell apical/basal polarity. The enriched cellular components mainly included the intracellular protein-containing complex, centrosome, condensed chromosome, preribosome, large subunit precursor, replication fork, nuclear speck, and ficolin-1-rich granule. The enriched molecular functions mainly included catalytic activity acting on nucleic acid, hyaluronoglucosaminidase activity, pyrophosphatase activity, phosphotransferase activity, histone binding, DNA-binding transcription activator activity, ubiquitin-conjugating enzyme binding, exoribonuclease activity, and producing 5′-phosphomonoesters. The KEGG enrichment analysis results are shown in Fig. 11 and include RNA degradation, glycosaminoglycan degradation, biosynthesis of cofactors [22], N-glycan biosynthesis, cell cycle, purine metabolism, and biosynthesis of amino acids. These results indicate that HYAL3 is closely associated with processes related to the regulation of tumor cell differentiation and growth.

### HYAL3 expression correlates with immune cell infiltration in BLCA

**Fig. 12:**
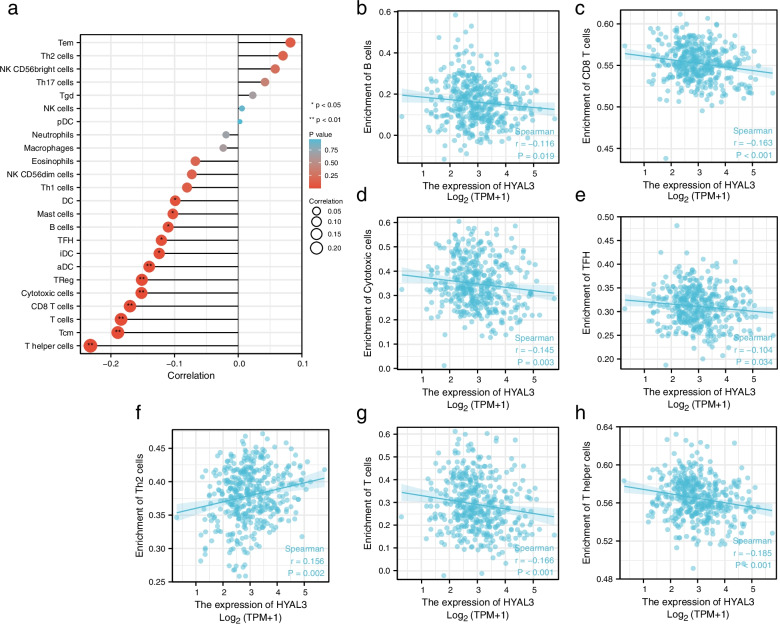
The correlation between *HYAL3* expression and several types of infiltrating immune cells in bladder cancer (BLCA) patients. **a** Summary. **b** B cells. **c** CD8 T-cells. **d** Cytotoxic cells. **e** T follicular helper cells. **f** Th2 cells. **g** T cells

We analyzed the associations between various types of infiltrating immunocytes and *HYAL3* expression in BLCA patients. The results are shown in Fig. 12. *HYAL3* expression was negatively associated with B cells (*R* = -0.116, *P* = 0.019), CD8^+^ T cells (*R* = -0.163, *P* < 0.001), cytotoxic cells (*R* = -0.143, *P* = 0.003), T follicular helper cells (*R* = -0.104, *P* = 0.034), T helper (Th) 2 cells (*R* = 0.156, *P* = 0.002), T cells (*R* = -0.166, *P* < 0.001), and Th cells (*R* = -0.185, *P* < 0.001).

To further explore the potential function of HYAL3 in various infiltrating immunocytes in BLCA, we analyzed the GEPIA and TIMER databases regarding the associations between *HYAL3* and several immunologic marker sets with the corresponding signs of different types of immunocytes (Table 3). The results indicate that the *HYAL3* expression was associated with Th1, Th2, Th9, Th22 functional T cells, M1 macrophages, and neutrophils in BLCA, suggesting that HYAL3 might be associated with immune responses in BLCA.

**Table 3:** Correlation analysis between *HYAL3* and markers of immunocytes in TIMER and GEPIA

Cell type	Gene marker	None		Purity		Tumor		Normal	
		Cor	*P*	Cor	*P*	Cor	*P*	Cor	*P*
B cells	*CD19*	−0.105	*	− 0.128	*	− 0.091	0.069	0.24	0.32
	*CD20 (KRT20)*	0.056	0.258	0.067	0.198	0.093	0.061	0.43	0.064
	*CD38*	−0.083	0.094	−0.097	0.063	− 0.079	0.11	0.69	0.097
CD8^+^ T cells	*CD8A*	− 0.084	0.089	− 0.089	0.115	− 0.042	0.4	0.16	0.51
	*CD8B*	−0.065	0.188	−0.055	0.292	0.01	0.84	0.15	0.54
Tfh	*BCL6*	−0.137	**	−0.153	**	−0.076	0.13	−0.37	0.12
	*ICOS*	−0.155	*	−0.122	*	−0.069	0.17	0.25	0.31
	*CXCR5*	−0.084	0.09	−0.111	*	0.19	0.066	0.31	0.2
Th1	*T-bet (TBX21)*	−0.099	*	−0.11	*	−0.063	0.21	0.28	0.24
	*STAT4*	−0.133	**	−0.148	**	−0.069	0.17	0.2	0.42
	*IL12RB2*	−0.039	0.4	−0.02	0.7	−0.017	0.73	0.24	0.32
	*WSX1 (IL27RA)*	0.183	**	0.23	***	0.25	***	0.15	0.55
	*STAT1*	−0.091	0.06	−0.099	0.06	−0.055	0.27	0.34	0.15
	*IFN-γ (IFNG)*	−0.061	0.218	−0.053	0.3	−0.013	0.79	−0.18	0.45
	*TNF-α (TNF)*	0.021	0.7	0.04	0.4	−0.024	0.63	0.043	0.86
Th2	*GATA3*	−0.005	0.923	0.006	9.14 × 10^−4^	− 0.00084	0.99	0.25	0.3
	*CCR3*	0.104	*	0.102	*	0.055	0.27	−0.17	0.49
	*STAT6*	−0.164	***	−0.165	***	−0.14	**	−0.16	0.5
	*STAT5A*	0.026	0.6	0.03	0.56	0.013	0.79	−0.45	0.051
Th9	*TGFBR2*	0.006	0.9	0.005	0.9	0.11	*	−0.48	*
	*IRF4*	−0.092	0.06	−0.118	*	−0.089	0.073	0.25	0.31
	*PU.1 (SPI1)*	−0.05	0.31	−0.069	0.18	0.055	0.27	−0.052	0.83
Th17	*STAT3*	−0.052	0.299	−0.07	0.181	0.0048	0.92	−0.32	0.18
	*IL21R*	−0.071	0.154	−0.088	0.09	−0.023	0.64	0.18	0.46
	*IL23R*	−0.072	0.147	−0.087	0.09	−0.031	0.53	0.14	0.57
	*IL17A*	−0.059	0.236	−0.048	0.36	0.022	0.66	−0.2	0.41
Th22	*CCR10*	0.068	0.17	0.075	0.15	0.11	*	−0.17	0.48
	*AHR*	−0.056	0.26	−0.043	0.411	0.009	0.86	0.16	0.53
Tregs	*FOXP3*	−0.107	*	−0.112	*	−0.045	0.37	−0.0053	0.98
	*CD25 (IL2RA)*	−0.066	0.186	−0.076	0.146	−0.006	0.9	−0.15	0.54
	*CCR8*	−0.118	0.018	−0.126	0.016	−0.066	0.18	0.0081	0.97
T cell exhaustion	*PD-1 (PDCD1)*	−0.095	0.055	−0.098	0.059	−0.056	0.26	0.25	0.31
	*CTLA4*	−0.118	*	−0.119	*	−0.088	0.078	0.24	0.33
	*LAG3*	−0.037	0.459	−0.02	0.7	−0.011	0.82	0.08	0.74
	*TIM-3 (HAVCR2)*	−0.056	0.257	−0.067	0.198	0.0077	0.88	−0.11	0.65
Macrophages	*CD68*	−0.08	0.1	−0.094	0.06	0.021	0.67	−0.041	0.87
	*CD11b (ITGAM)*	0.013	0.788	0.022	0.676	0.017	0.73	−0.36	0.13
M1	*INOS (NOS2)*	0.053	0.289	0.071	0.174	−0.0016	0.97	0.19	0.44
	*IRF5*	0.064	0.197	0.077	0.142	0.12	*	0.36	0.13
	*COX2 (PTGS2)*	−0.04	0.416	−0.043	0.413	0.079	0.11	−0.22	0.36
M2	*CD163*	0.018	0.716	0.025	0.628	0.04	0.43	−0.38	0.11
	*ARG1*	−0.04	0.42	−0.015	0.77	0.034	0.5	−0.09	0.71
	*MRC1*	0.005	0.922	0.012	0.814	0.02	0.68	−0.45	0.052
	*MS4A4A*	−0.029	0.562	−0.033	0.529	0.042	0.4	−0.48	*
TAMs	*CCL2*	0.025	0.618	0.047	0.369	−0.018	0.71	−0.26	0.28
	*CD80*	−0.05	0.31	−0.048	0.363	−0.038	0.45	0.14	0.57
	*CD86*	−0.085	0.08	−0.104	0.045	−0.04	0.43	0.012	0.96
	*CCR5*	−0.054	0.278	−0.058	0.264	−0.028	0.58	0.29	0.24
Monocytes	*CD14*	−0.01	0.834	−0.015	0.781	0.075	0.13	−0.27	0.27
	*CD16 (FCGR3B)*	0.014	0.785	0.045	0.385	0.057	0.26	−0.13	0.59
	*CD115 (CSF1R)*	−0.057	0.249	−0.078	0.135	0.072	0.15	−0.37	0.12
Neutrophils	*CD66b (CEACAM8)*	0.035	0.475	0.04	0.44	0.043	0.38	0.33	0.16
	*CD15 (FUT4)*	0.132	**	0.13	*	0.12	*	0.069	0.78
	*CD11b (ITGAM)*	0.013	0.788	0.022	0.676	0.017	0.73	−0.36	0.13
Natural killer cells	*XCL1*	−0.09	0.07	−0.069	0.184	−0.055	0.27	−0.11	0.64
	*CD7*	−0.058	0.247	−0.051	0.331	0.0027	0.96	0.14	0.56
	*KIR3DL1*	−0.059	0.236	−0.056	0.28	0.021	0.68	0.29	0.24
Dendritic cells	*CD1C (BDCA-1)*	−0.151	**	−0.176	**	−0.037	0.45	0.64	**
	*CD141(THBD)*	−0.084	0.08	−0.093	0.07	−0.042	0.4	0.0052	0.98
	*CD11c (ITGAX)*	−0.012	0.8	−0.012	0.805	−0.029	0.56	0.19	0.44

*Tfh* Follicular helper T cells, *Th* T helper cells, *Tregs* Regulatory T cells, *TAMs*: Tumor-associated macrophages. None, Correlation without adjustment. Purity, Correlation adjusted by purity. Cor, R value of Spearman’s correlation. *P < 0.05; **P < 0.01; *** *P* < 0.001

## Discussion

In this study, we explored the prognostic importance of HYAL3 in BLCA, with regard to the biological processes, molecular functions, cellular components, and potential signaling pathways. We found that the *HYAL3* expression level could assist in the diagnosis of BLCA and that it further predicted the OS of BLCA patients. In addition, we explored the correlation between *HYAL3* expression and the type of infiltrating immunocytes and found that HYAL3 was associated with glycosaminoglycan degradation. According to the protein–protein interaction network analysis results, it was found that the HYAL3-related gene *SPAG9* was associated with immune response [23]. In addition, *IDUA* is a novel glycolysis-related gene that is associated with the immune microenvironment in renal cell carcinoma [24]. Moreover, *ELN* has been reported to be an immune-related gene in BLCA [25]. *HYAL3* was associated with several GO functions, including androgen receptor signaling, response to oxidative stress, and negative regulation of the target of rapamycin signaling, the latter of which might correlate with immunocyte infiltration [26–28]. In the TCGA-BLCA database, we found that *HYAL3* was associated with several types of infiltrating immunocytes, including DCs, mast cells, B cells, T follicular helper cells, interdigitating DCs, activated DCs, regulatory T cells, cytotoxic cells, CD8^+^ T cells, T cells, central memory T cells, and Th cells. Some of these cells have been shown to participate in the pathogenesis of BLCA [29–31]. These results shed light on new pathways through which HYAL3 contributes to BLCA carcinogenesis, potentially by regulating immunocyte infiltration.

BLCA is a high-risk malignancy [32], and an early diagnosis and prognostic prediction are important. Existing prediction models are mostly based on mRNAs, miRNAs, or clinical characteristics [33–35]. In addition, infiltrating immunocytes play an important role in regulating BLCA pathogenesis [36]. BLCA has a relatively high tumor mutational burden and is responsive to immunotherapeutic approaches such as Bacillus Calmette–Guerin immunotherapy. Therefore, BLCA is often regarded as an immunogenic tumor [37]. Evidence indicates that a high density of tumor-infiltrating CD8^+^ T cells is a favorable prognostic factor among BLCA patients, whereas programmed death-ligand 1 expression and tumor-associated macrophages are unfavorable features [38–40]. Consequently, the exploration of the association between BLCA and tumor-infiltrating immunocytes might be therapeutically beneficial.

*HYAL3* belongs to the group of genes located on chromosome 3p21.3 that exhibit an association with tumor suppression; furthermore, the expression of specific transcript variants may parallel the tumor status [13]. Prior research has suggested that HYAL3 promotes tumor growth in colorectal cancer [41]. A well-coordinated HA turnover participates in biological processes including cellular proliferation, migration, and adhesion. Moreover, HA may assist in escaping from immune surveillance [14]. HYAL has been shown to accumulate in the tumor microenvironment and to correlate with tumor development and invasion [42]. Researchers also have tried to use HYAL to enhance the antitumor efficacy of tumor vaccines in vivo. HYAL has been demonstrated to potentially increase the permeability of tumor tissues by breaking down HA in the tumor ECM, enabling effective immune responses to be mounted for controlling or eliminating malignant cells [43]. Additionally, during the treatment of glioblastoma multiforme, researchers have found that using HYAL-expressing oncolytic adenovirus ICOVIR17 can degrade HA in tumor cells, subsequently modifying the immunologic landscape of the tumor microenvironment [44]. Altogether, these results support our findings that *HYAL3* may participate in encoding HYAL and regulating tumor microenvironment-infiltrating immunocytes.

Several limitations existed for this study. For example, the online databases contained multiple issues that warrant consideration, including racial restrictions and the numbers of BLCA cases and normal tissues. A longer follow-up period and an increased number of patients in future studies are still needed.

## Conclusion

A higher *HYAL3* expression level might predict a shorter OS among BLCA patients. *HYAL3* was additionally associated with several types of infiltrating immunocytes in BLCA, including Th cells, T cells, CD8^+^ T cells, cytotoxic cells, B cells, etc. These data indicate that HYAL3 might serve as a biomarker for BLCA diagnosis and treatment in the future. Nevertheless, more research is needed to verify the biological functions of HYAL3 in BLCA.

## Supplementary Information

**Additional file 1.**

## Data Availability

The data used in this study are freely available from the TCGA dataset (https://portal.gdc.cancer.gov/projects/TCGA-BLCA) and the GEO dataset (http://www.ncbi.nlm.nih.gov/geo/). The authors did not have special access privileges.

## Abbreviations

BLCA: Bladder cancer
HA: Hyaluronan
HYAL3: Hyaluronidase 3
ECM: Extracellular matrix
TIMER: Tumor Immune Estimation Resource
TCGA: The Cancer Genome Atlas
ROC: Receiver-operating characteristic
OS: Overall survival
GO: Gene Ontology
KEGG: Kyoto Encyclopedia of Genes and Genomes
AUC: Area under the curve
CI: Confidence interval
